# Searching for Digital Technologies in Containment and Mitigation Strategies: Experience from South Korea COVID-19

**DOI:** 10.5334/aogh.2993

**Published:** 2020-08-31

**Authors:** Kyungmoo Heo, Daejoong Lee, Yongseok Seo, Hyeseung Choi

**Affiliations:** 1 Moon Soul Graduate School of Future Strategy, Korea Advanced Institute of Science and Technology (KAIST), Yousung, Daejeon, KR; 2 Development Finance Division, Korea Ministry of Economy and Finance, Government Complex, Sejong, KR; 3 Division of International Studies, Hanyang University, Sageun, Seongdong, Seoul, KR

## Abstract

**Background:**

Korea has achieved health policy objectives in pandemic management so far, namely minimizing mortality, flattening the epidemic curve, and limiting the socio-economic burden of its measures. The key to the Korean government’s success in combating COVID-19 lies with the latest digital technologies (DTs). The prompt and effective application of DTs facilitates both containment as well as mitigation strategies and their sub-policy measures.

**Methods:**

This article uses an experiential analysis based on an exploratory case study – analysis on field applications of the government’s interventions. Information is collected by qualitative methods such as literature analysis, meeting materials, and a review of various government reports (including internal ones) along with academic and professional experiences of the authors.

**Findings:**

The article presents the unique Korean health policy approaches in the COVID-19 crisis. First, DTs allow the Korean government to embrace various policy measures together listed in containment strategy, namely altering and warning, epidemiological investigation, quarantine of contacts, case-finding, social distancing, and mask-wearing. Second, DTs allow Korea to integrate containment and mitigation strategies simultaneously. Along with the above measures in containment, healthcare service, medical treatment, and prophylaxis (presymptomatic testing) within mitigation are utilized to prevent a COVID-19 spread.

**Conclusions:**

Korea develops DTs in an integrated manner in the early pandemic stage under strong and coordinated government leadership. Above all, the DTs’ functions in each pandemic developmental stage are continuously upgraded. Instead of prioritizing policy measures or strategies, therefore, Korea can implement diverse policies simultaneously by integrating DTs effectively. During the COVID-19 outbreak, DTs work as the enablers to connect these two strategies and their measures in Korea.

**Recommendations:**

DTs should be at the center of the disaster management paradigm, especially during a pandemic. DTs are facilitators and integrators of containing and mitigating strategies and their policy measures.

## 1. Introduction

South Korea (hereinafter Korea) faces a surge of 2019 coronavirus disease (COVID-19) outbreak where the number of new coronavirus cases increased exponentially peaking on February 29, 2020, with a record of 909 new infections [1]. Despite its continued appearance of small confirmed cases recently, Korea was able to successfully flatten the COVID-19 curve in just 20 days [2]. It is pandemic management health policies that were rather different from the international recommendations, namely containment first then mitigation [3,4]. What is interesting is that Korea did not prioritize policy measures in either containment or mitigation strategy or choose one strategy from mitigation and containment. All measures and strategies were integrated and implemented under strong and coordinated government leadership.

Based on international practices of containment and mitigation strategies and their measures, this article describes policies, actors, and processes of the Korean pandemic management along with its enabling digital technologies (DTs). For this objective, it then attempts to address the following questions. First, how does Korea embrace various responding measures listed in containment strategy while minimizing opportunity cost coming from prioritization of these measures? Second, how does Korea successfully implement both containment and mitigation strategies simultaneously?

This article first explains methods and theoretical background followed by the Korean comprehensive policy measures using DTs and other innovative policies between March 2020 – June 2020. Then, it will delve into how DTs play a role in containing and mitigating COVID-19 by enabling, facilitating, and integrating all relevant measures and strategies. Lastly, this article offers recommendations for other countries.

## 2. Methods and Theoretical Background

**Figure 1:**
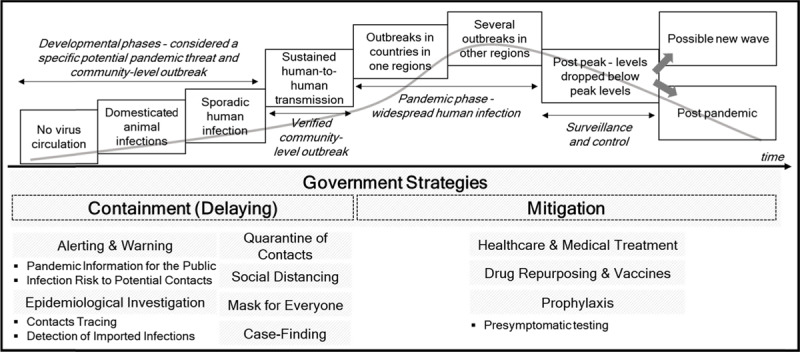
Phases of Pandemic with Measures in Containment and Mitigation. * Reference: [4, p.11 & p.24; 8].

In a pandemic outbreak situation, policy objectives of a government are namely to minimize mortality, to flatten the epidemic curve, and to limit the socio-economic burden of its measures [5,6,7]. A government utilizes a range of policy measures to achieve these objectives. Both containment (delaying) and mitigation strategies are mostly introduced as the government’s policy interventions. The World Health Organization defines containment as a means to prevent or delay the spread of infection while mitigation as a means to reduce the impact of the pandemic [3]. The details of both containment and mitigation strategies are depicted in Figure 1 [3,8].

**Figure 2:**
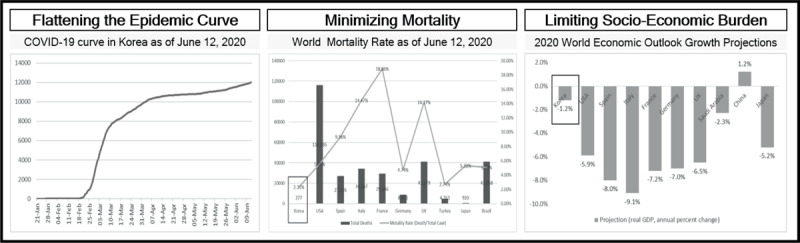
Achievement Level of Korea in COVID-19 Policy Objectives. * Reference: [1]; * Reference: [13]; * Reference: [14].

Various pieces of literature and reports in epidemiology, infectious diseases, and public health policy have researched about containment and mitigation strategies in terms of their applicable conditions [9,10], effectiveness [4], and sequences [11]. These papers also suggest a government prioritize measures or select a certain one listed in mitigation and/or containment strategies based on the phase and severity of a pandemic (refer to Figure 1 for the phase of pandemic and relevant strategies) [3]. Because of limited resources and time, putting all measures or strategies into certain plays assumes to be challenging [12]. Furthermore, focusing heavily on one measure may jeopardize other efforts of a government [5]. For example, continued border control may cause the tourism industry to face its downturn. As seen on Figure 2 [1,13,14], however, Korea is recognized as achieving major policy objectives in pandemic management by applying all these strategies and measures simultaneously as of June 12, 2020.

This article uses an experiential and rather reflective analysis [15,16,17] based on an exploratory case study [18,19] – qualitative analysis on field applications of the government’s interventions, namely the DT-based policy measures of Korea. We are taking into account the research questions of this article – ‘why’ and ‘how,’ it is critical to provide detailed but underlying information and meaningful implications. Therefore, this analysis supports understanding the in-depth context in applying them and making policy practitioners for timely decisions within the uncertainty of the global pandemic. For scholars, a result of this analysis can be a trigger point to interact between interested actors and implicate multiple interpretations with respect to meaning, relevance, and importance, as a potential to shape future research.

This article delivers unique but practical implications for both health policy practitioners and scholars with the Korean lessons learned written in two English written policy reports. These reports, published from the Government of Korea, namely Tackling COVID-19 Health, Quarantine and Economic Measures: Korean Experience [20] and Flattening the Curve on COVID-19: How Korea Responded to a Pandemic Using Information and Communication Technologies [21] are the foundation of this article. They have received wider recognition from foreign countries and international communities such as the World Bank and the United Nations. So far, the total four reports have been published, and the 5^th^ one is about to be published at the end of August 2020. Our attempt is to bring these rather intuitive governmental reports into the academic realm. In addition, to enrich the contents, additional information is collected by qualitative methods such as literature analysis, meeting materials, and a review of various government reports (including internal ones) along with academic and professional experiences of the authors.

## 3. Result

### 3.1. Policy Measures for Containment Strategy

#### 3.1.1. Timely Delivery of Public Information

*Cellular Broadcasting Service (CBS)*. CBS enables the Korean government to transmit emergency alert text messages on the COVID-19 outbreak. Instead of sending a message to each individual, CBS allows the government to broadcast an emergency situation from the mobile telecom base stations. Because of this broadcasting approach, CBS can warn individuals and send short emergency alerts and guidelines for the public when they enter into a disaster area. National Disaster and Safety Status Control Center of the Ministry of the Interior and Safety (MOIS) is in charge of the system [21], but provincial and municipal governments can also send alert messages to their local residents without approval from the MOIS. With support from the mobile telecom carriers, CBS sends messages without a delivery bottleneck when an emergency occurs. It is because CBS has its own channel for texting messages and does not use the general SMS text messaging system. Moreover, the government made an agreement with the three largest mobile carriers in August 2019 about sharing their own designated frequency channel for an emergency alert and a phone call.

*Artificial-Intelligence (AI)-based Chatbot in Municipal Authorities*. The Sungnam municipal government provides the AI-based care call service to those under active monitoring [21]. The AI chatbot calls twice a day to check whether they have a fever or any respiratory symptom and automatically send the check-up results to the public health clinic via email. The service also includes the provision of AI speakers at a patient’s home in order to offer two-way communication on COVID-19 situation [22]. It answers questions of individuals and automatically informs the latest COVID-19 developments such as the number of the confirmed cases and deaths, prevention guidelines, and information on mask-availability in neighboring areas.

#### 3.1.2. Government Transition to Smart Life

*Smart Working*. The Korean government launched a website to provide the private companies’ software solutions of remote working to private and public organizations. The website includes information on solution providers such as contact numbers and featured functions as well as the price of their products [21]. After a joint survey with the Korea Software Industry Association, the government identifies the demand for solutions and classifies more than 140 solutions by the business sector [21]. To remove the burden of applying the government remote working scheme within a short period of time, the government also offers free solutions and related infrastructural and technical supports. Moreover, the government takes the initiative and an exemplary role in remote working. For example, municipalities adopt real-time virtual meetings along with flexible working hours to minimize contacts. 30% of all central government officers enter into the mandatory smart working scheme [22]. To maintain the required level of security in the government affairs, the government establishes Global Virtual Private Network (the government’s intranet that enables access outside their offices) and sets up G-Drive (the government’s cloud storage system) [22].

*Remote Education*. As of April 20, 2020, all Korean students start mandatory online public education [21]. It is the outcome of the government’s prompt action in setting responding policies and DT infrastructures. First, to support students with limited digital access in the vulnerable class, the government lends smart devices such as computers and smart tablets that donated from the private sector. Second, the government exempts smartphone data and subscription charges when students access the Korea Educational Broadcasting System website. Third, for higher education such as universities and colleges, the government creates online simulation software and platform where students can experience hands-on learning content for 23 high-demand classes (e.g., quantum chemistry).

#### 3.1.3. Development of Public Mobile Applications

**Figure 3:**
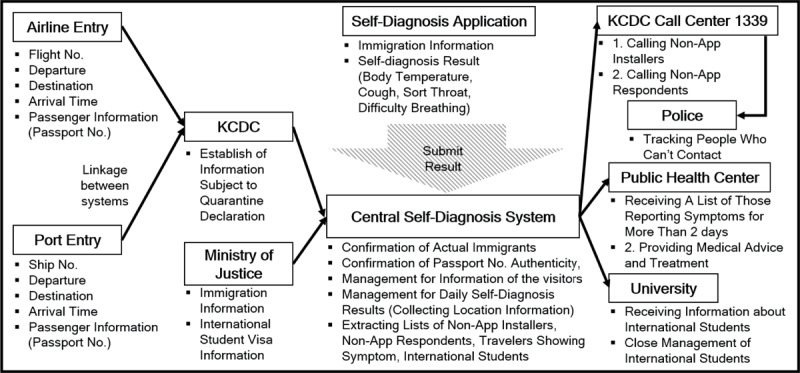
Process of Self-Diagnosis. * Reference: [22]. ** Abbreviation: KCDC, Korea Centers for Disease Control and Prevention.

*Self-Diagnosis Mobile Application.* The Korean government develops the mobile diagnosis application to monitor any possible COVID-19 symptoms of inbound passengers and to provide them with prompt medical advice. The application can be downloaded easily at the airport/harbor immigrations. A passenger is required to install it when submitting his/her information at the immigrations [20]. All inbound passengers must report their health conditions through the diagnosis application once a day during their 14 days of mandatory self-quarantine [21,22]. Submitted data is collected by the central diagnosis system under strong privacy protection. When any suspicious symptom appears, passengers are encouraged to seek medical advice first through call centers operated by the Korea Centers for Disease Control and Prevention (KCDC), or at COVID-19 screening centers [1]. Symptoms are crossed checked with their immigration data (e.g., nationality and a country visited) and daily health information collected through the application before being sent to public health clinics. Information on those with symptoms for more than two days is transferred to local governments. Then, corresponding public health clinics provide medical advice, testing, and instructions on how to be treated. Figure 3 describes the process of self-diagnosis and the roles of relevant public administrations and local governments.

**Figure 4:**
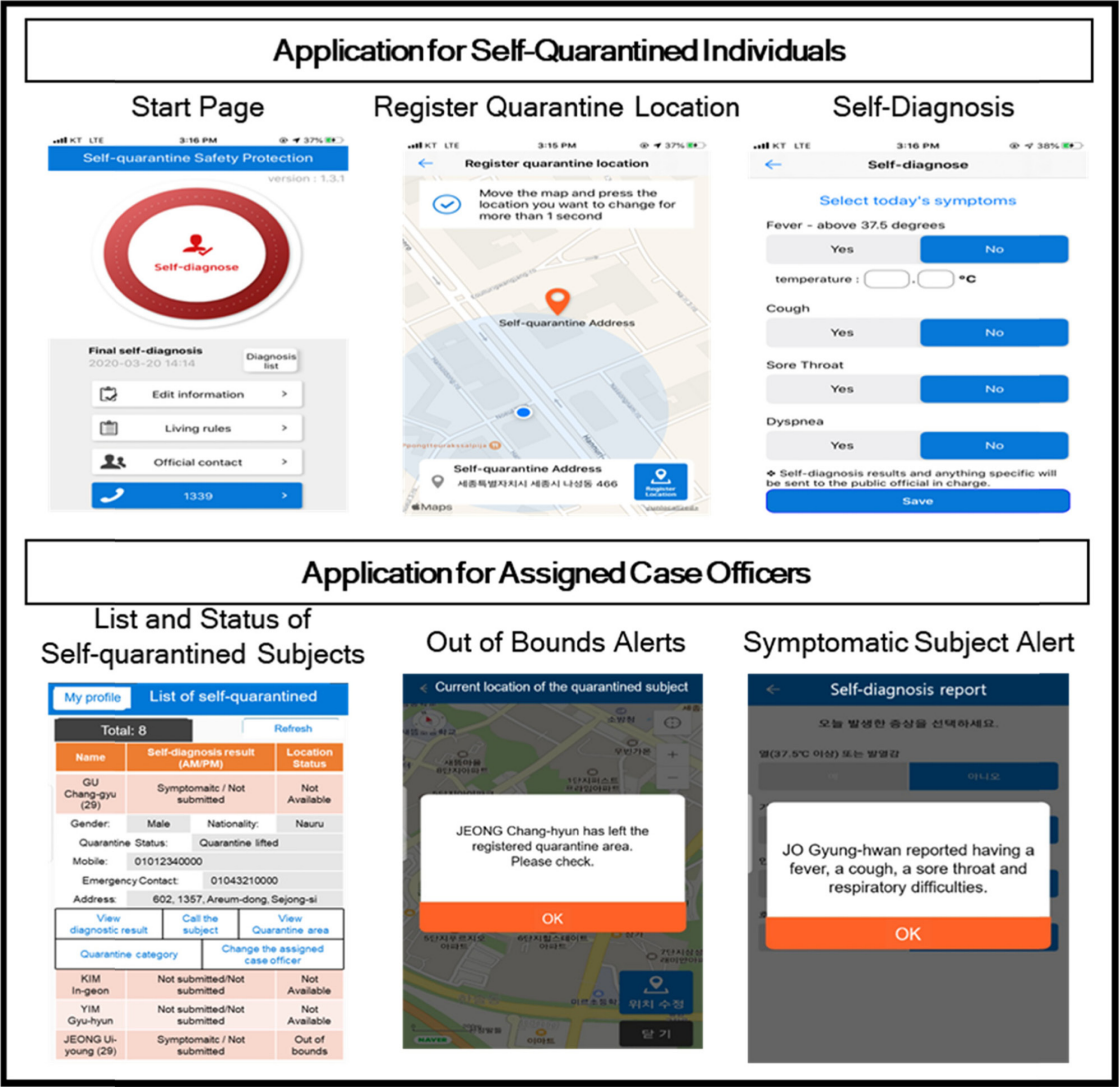
Self-Quarantine Safety Applications. * Reference: [21].

*Self-Quarantine Safety Mobile Application.* The Korean government develops the quarantine application to effectively monitor those under self-quarantine. This application has three key functions: submitting a self-diagnosis to their assigned government officers, a GPS-based location tracking to prevent possible violations of self-quarantine orders, and providing necessary self-quarantine guidelines [20]. Those under self-quarantine use the application twice a day to monitor four symptoms: fever, cough, sore throat, and respiratory difficulties [21]. Once submitted, the self-diagnostic data are automatically transferred to an assigned case-officer. A case-officer is notified when individuals leave their self-quarantine area, and an abnormal result of a self-diagnosis is detected. Two types of quarantine applications are developed: one for the users under self-quarantine and the other for government case-officers. Figure 4 shows visual images of two applications.

#### 3.1.4. Data-based Government’s Epidemiological Investigation

*Smart Quarantine Information System (SQIS)*. Tracing inbounded passengers and their potential contacts is an important part of an epidemiological investigation, especially when an infection risk from a certain COVID-19-rampant country is high. The Korean government introduces SQIS which utilizes the overseas roaming data information to identify inbound passengers from high-risk regions and to monitor them during the latent period of the infection [20]. SQIS is managed by epidemiological investigative teams organized promptly by the director of KCDC, mayors, and governors based on the Infectious Disease Control and Prevention Act [20]. To increase the accuracy of contact tracing, SQIS integrates information from the National Police Agency, mobile carriers, and credit card companies.

*Epidemiological Investigation Support System (EISS)*. When daily confirmed cases reach a peak, epidemiological investigators underwent a heavy workload. The Ministry of Land, Infrastructure, and Transport and KCDC together develop a computer-aided system, namely EISS, to analyze a large amount of data. EISS supports epidemiological investigators in quickly identifying the transmission routes and places that the confirmed cases visited [20]. First, EISS simultaneously collects various types of real-time data such as individual GPS information from a mobile phone and credit-card transaction history. EISS collects data within 10 minutes and promptly conducts a spatial-temporal analysis. Second, public health officials use data in EISS when they cross-check the epidemiological interview responses of the patients. Third, the EISS’s big data analysis allows officials to have real-time data feeds on confirmed cases, including their whereabouts and the time spent on each location. Based on these multiple data points, the system can detect cluster infection and identify the source of transmission.

### 3.2. Measures for both Mitigation and Containment Strategies

#### 3.2.1. Remote Public Healthcare with Telemedicine Service

The Korean government temporarily permits doctors to engage in telemedicine. This measure has two purposes [20]. The first purpose is to avoid group contagion in virus vulnerable facilities such as medical institutions and nursing homes. The second is to prevent further spread of infection to other provinces. For example, in Daegu City, where the largest confirmed cases are detected, local hospitals link their network with their well-known but far-away mother hospitals by synchronizing electronic hospital information systems in real-time [21]. Medical questionnaires filled out by patients and X-ray scans are uploaded on the integrated system. Radiologists from mother hospitals which is hundreds of kilometers away from Daegu city, analyze uploaded medical scans, and record a diagnosis into the database. If necessary, local hospitals conduct patients’ medical examinations and treatments through video conferences with mother hospitals. When symptoms of confirmed patients are aggravated, local hospitals transfer them to designated COVID-19 treatment hospitals.

For mild and presymptomatic patients, hospital nurses use video calls in smartphones to check their daily conditions and further development of symptoms [20]. If patients show symptoms unrelated to COVID-19, nurses guide them to visit the government-designated National Security Hospitals that separate treatment processes of respiratory from non-respiratory patients to prevent in-hospital infection of COVID-19 to other patients. This separation process comes from the lessons learned from the Middle East Respiratory Syndrome outbreak in 2015 [23]. At that time, unseparated and unselected in-hospital contacts of confirmed and presymptomatic patients with normal patients triggered a surge of in-hospital infections in few largest hospitals in Korea.

#### 3.2.2. Information Sharing with Open Public Data

*Government-Corporation-Researchers Network*. The Korean government establishes a network composed of relevant stakeholders to fight against COVID-19. While protecting personal information, the government shares different levels of medical and epidemiological data with researchers for immediate R&D purposes [21]. For example, KCDC shares data of confirmed cases with the network for predictive research. By partnering with the largest mobile carrier in Korea, the government also provides researchers with data on the levels of foot traffic and international roaming for free. Such support is useful for forecasting the macroscopic spread of COVID-19 and evaluating the effectiveness of policy responses.

*Mobile Applications by Partnership between Government and Private Sectors*. Based on information released by KCDC, various COVID-19 related mobile applications are introduced to the public [21]. First, startups develop telemedicine service applications. These applications offer patients access to diagnosis, remote treatment, and prescriptions for suspected coronavirus symptoms. When application users are suspected of being infected with COVID-19, the applications automatically connect users to the government emergency hotline. In detail, the applications help patients to fill in medical questionnaires provided by COVID-19 screening hospitals prior to their hospital visits. Therefore, the applications contribute to minimizing the actual time patients spend at the hospitals, ultimately reducing the risk of in-hospital contagion.

There are applications that are widely used by the public. For example, there are applications that inform the public of the travel routes of confirmed patients [21]. Some applications calculate a mix of risk factors in surrounding areas when users enter their commuting routes. If users record their own routes, applications compare users’ paths with those previously taken by confirmed cases or new patients diagnosed with COVID-19. If users were at the same place as a confirmed patient at a similar time, applications suggest users conduct a presymptomatic test by providing information on when and where testing is available. In addition, there are applications that depict the number of public-procured face masks available at a given location in real-time. The availability status of public masks is illustrated in different colors: green (more than 100), yellow (between 30 and 99), red (below 30), and grey (none available) [21]. Information on mask sellers is automatically updated in accordance with data publicly released by the National Information Society Agency [21].

#### 3.2.3. Innovation by Government’s Fast Approval and Immediate Funding

*Agile Governmental Actions on COVID-19 Diagnostic Kit*. The government’s agile responses toward emergency approvals and immediate R&D fundings enable Korea to conduct COVID-19 tests as fast and many as possible. On February 4, only three weeks after the release of the COVID-19 genetic sequence, the government granted emergency use authorization for test kits [20]. The government continues to increase the number of public testing institutions and private test kit manufacturers. As of April 15, 2020, five diagnostic reagent companies have obtained emergency use approval in producing RT-PCR reagents, which are the chemical substance used in COVID-19 testing [20]. Moreover, the government’s continued funding on infectious disease R&D enables Korea to play a major role in eliminating uncertainties in the early stages of the virus spread. Three out of the above five test kit-manufacturing companies have received government funding [21] since the outreak of the Middle East Respiratory Syndrome outbreak in 2015. Their R&D capabilities in DTs, AI, and high-performance computing capacity contribute to swift product development and raise the daily testing kit production.

*AI Installation for Chest Radiography Detection*. Amid the rapid spreads of COVID-19, it is important for the Korean government to quickly screen patients and selectively accommodate patients with severe symptoms. The experimental AI-based treatment is then introduced and applied to the field of battle against COVID-19 to complement a lack of healthcare workers and a shortage of hospital beds. For example, some local governments install AI devices for chest radiography detection at public health centers and local hospitals to relieve the burden of healthcare workers and to improve the efficiency of diagnosis [21]. AI quickly learns, detects, and analyzes a large-scale chest X-ray photographic data. AI reads X-ray imaging of a patient’s lung condition and detects abnormal findings such as pneumonia – a major symptom of COVID-19 patients. AI then identifies those requiring intensive care with high accuracy in just a few seconds. The Korean government supports these innovative AI-based testing. For example, the government announced fast-track approval and funding support for AI R&D companies responding to COVID-19 on March 12, 2020 [21]. The high-performance computing and infrastructure offers preferentially to businesses that develop AI algorithms related to COVID-19.

**Figure 5:**
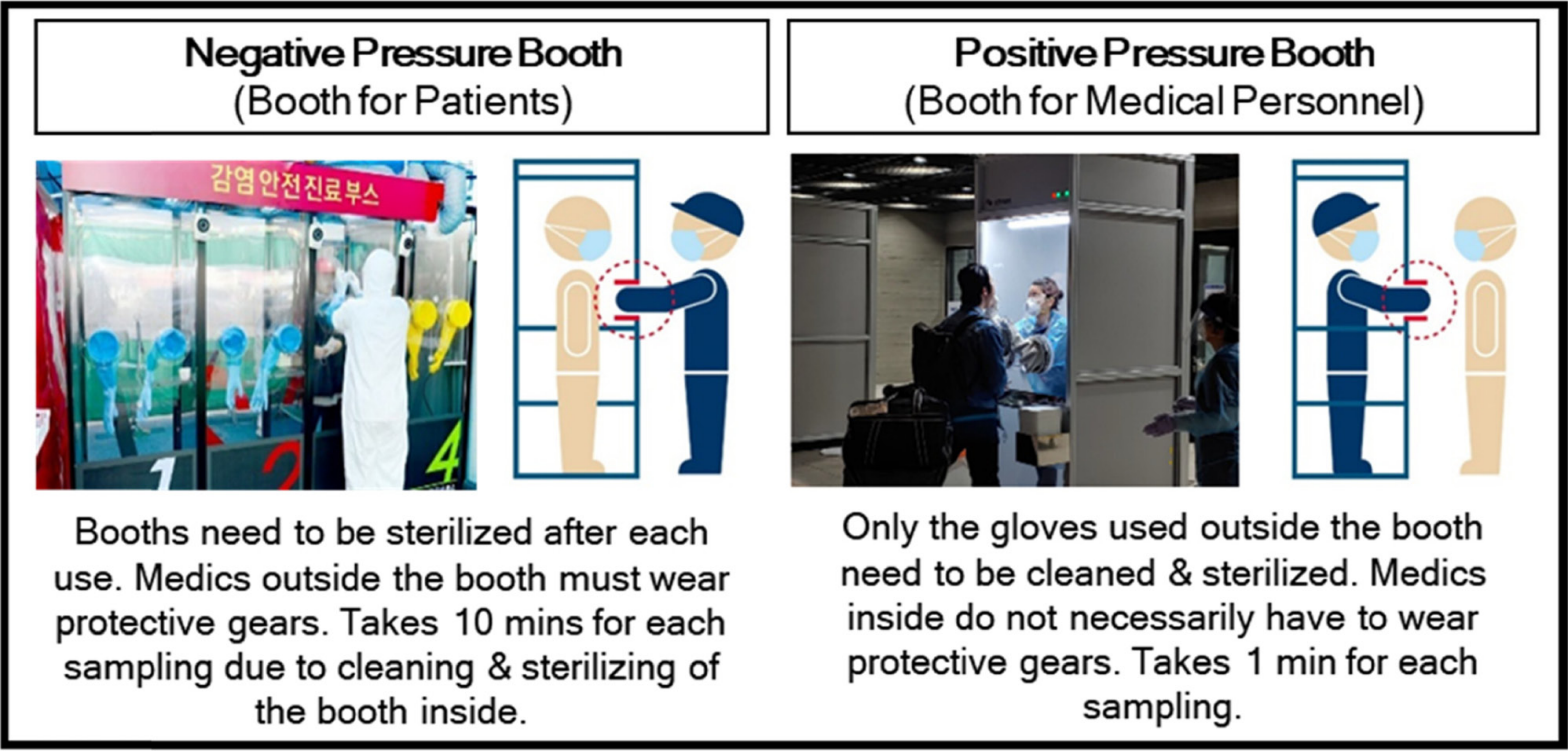
Two Types of Korea Walk-Through Testing Centers. * Reference: [20].

*Innovative Walk-through Testing Center (K-Walk-Thru)*. K-Walk-Thru is a test booth system adopted by Korea to quickly collect samples with minimal contact [20]. Despite its temporal operation, testing data are uploaded to the central system, and testing results are automatically shared with individuals tested. These innovative actions help minimize the risk of cross infections at the testing centers while maximizing daily testing capacity amid fast rates of new confirmed cases. The quick and safe K-Walk-Thru testing stations are available in two different types: negative pressure type and positive pressure type. They both consist of a single booth that separates healthcare professionals from individuals who are being tested. The only difference is in terms of who is inside the booth and how much time it takes for each sampling. The detailed explanation of K-Walk-Thru testing stations is in Figure 5.

## 4. Discussion

### Digital Technologies: Enabler, Facilitator, and Integrator

In the fight against the COVID-19 pandemic outbreak, the DT-based policy measures and strategies succeed Korea. The traditional concerns of both limited resources and uncertainties of a pandemic development in implementing measures for containment and mitigation together are offset by DTs. Korea does not prioritize measures or strategies, so to avoid the opportunity cost of choosing one instead of the other. It has shown that with the support of DTs, measures and strategies can be implemented simultaneously and successfully.

DTs embrace various government measures listed in a containment strategy. Korea introduces DTs in the early pandemic stage. By continuously upgrading the DTs’ functions in each pandemic developmental stage, Korea can implement these diverse measures simultaneously. Table 1 summarizes each DT-based measure in the mitigation strategy. First, CBS and AI Chatbots enable two-way communications between the government and the public. By sharing facts on the pandemic through CBS and AI Chatbot, the government informs practical guidelines on social distancing and wearing masks. Second, smart working and remote education systems support the implementation of strict social distancing measures and indirectly facilitate self-isolation. Third, the early installation of self-diagnosis mobile applications at the border control allows multiple measures at the same time – tracking inbound passengers, monitoring their potential infections, and encouraging self-isolation. Quarantine application is similar to self-diagnosis applications, but it focuses more on detecting infections and ultimately finding possible confirmed cases. Fourth, SQIS and EISS in epidemiological investigations support the public administration to trace contacts, find confirmed cases, and detect imported infections.

**Table 1:** Digital Technologies for Containment Strategy.

DTs in Korea		CBS	AI Chatbot	Smart Working	Remote Education	Diagnosis App.	Quarantine App.	SQIS	EISS

Measures									

Alerting and Warning	Pandemic Information for the Public	O	O						
	Infection Risk to Potential Contacts	O	O						

Epidemio-logical Investigation	Contacts Tracing		O			O	O	O	O
	Detection of Imported Infections		O			O		O	O

Quarantine of Contacts	Border Control and Tracking					O			
	Voluntary or Involuntary Isolation		O	O	O	O	O		

Case-Finding			O			O	O	O	O
Social Distancing		O	O	O	O			O	O
Mask-Wearing		O							

* Abbreviation: AI, Artificial intelligence; App.: Application; CBS, Cellular Broadcasting Service; DT, Digital technology; EISS, Epidemiological Investigation Support System; SQIS, Smart Quarantine Information System.

DTs integrate containment and mitigation strategies effectively. Korea implants both strategies in the early design stage of DTs. During the COVID-19 outbreak, DTs work as the enablers to connect these two strategies. Table 2 summarizes each DT-based measures in both containment and mitigation strategies. First, by applying remote public healthcare with telemedicine service at the early stage of the pandemic outbreak, the government contains diseases and relieves mild symptoms. These remote services screen the actual COVID-19 symptoms and provide prophylaxis for asymptomatic patient such as prescription of antibiotics and antiviral medicines at home. Consequently, it can prevent possible in-hospital infections. Second, a triangular cooperation network formed by the government, corporations, and researchers enables treatments that prevent something from happening, namely prophylaxis. The network suggests presymptomatic testing on individuals in a certain region and develops better healthcare solutions. These achievements are based on studies on the effectiveness of wearing-face masks, social distancing, and epidemiological forecasting. Moreover, mobile applications developed by the effective and prompt public-private partnership connect other containment measures with presymptomatic testings. Third, the government’s fast approval and immediate funding to COVID-19 diagnostic kit, installation of AI for chest radiography detection, and a new type of testing center help innovation of mitigation and containment strategies. These government’s actions fasten and widen the application of presymptomatic testing and indirectly support medical personnel to focus on the treatment by relieving them from the heavy workload of X-ray encryption.

**Table 2:** Digital Technologies for both Containment and Mitigation Strategies.

DTs in Korea		Remote Public Healthcare with Telemedicine Service	Government-Corporation-Researchers Network	Mobile Application by Public-Private Partnership	COVID-19 Diagnostic Kit	AI for Chest Radiography Detection	K-Walk-Thru Testing Centers

Measures							

**Containment Strategy**							

Alerting and Warning				O			
Epidemio-logical Investigation		O	O	O	O	O	O
Quarantine of Contacts		O					O
Case-Finding		O			O	O	O
Social Distancing		O	O	O		O	O
Mask-Wearing			O	O			

**Mitigation Strategy**							

Healthcare and Medical Treatment		O	O			O	
Drug Repurposing and Vaccines							
Prophylaxis (Pre-symptomatic Testing)		O	O	O	O	O	O

* Abbreviation: AI, Artificial intelligence; COVID-19, 2019 coronavirus disease; K-Walk-Thru, Korea walk-through.

## 5. Conclusion

### Disaster Management Paradigm with New Thought

This article presents the actual responses of the Korean government against COVID-19 using the latest DTs. It also discusses how Korea smartly fights against an invisible micro-pathogen. Korea has made a success in containing and mitigating COVID-19 so far. The traditional method of applying specific measures or strategies depending on the phase and development of the epidemic is not applied in Korea. Korea rather embraces measures for containment strategy and integrates containment and mitigation strategies effectively under DTs.

**Figure 6:**
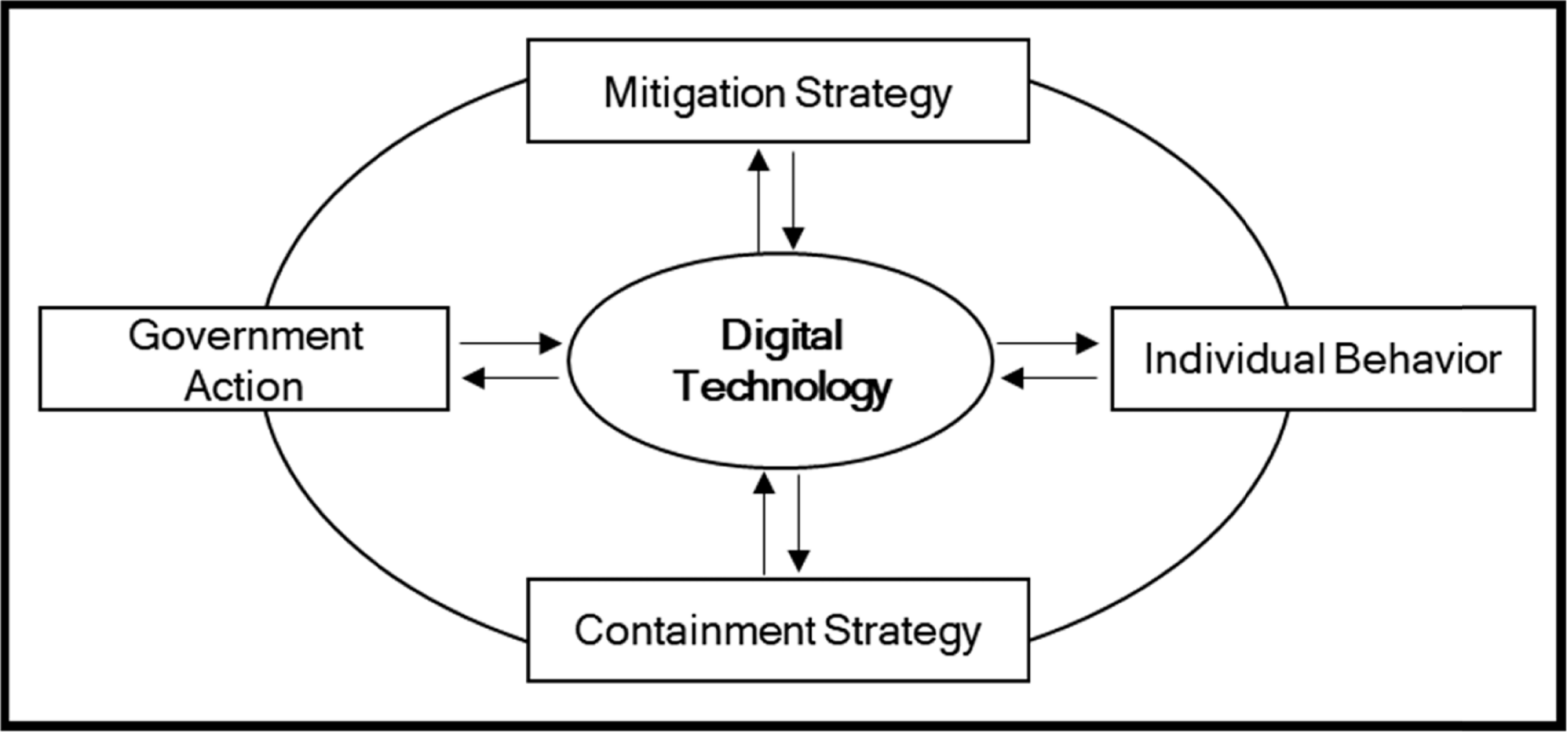
Digital Technology-Centered Epidemic and Pandemic Paradigm.

Based on the Korean experience, this article recommends changing the disaster management paradigm, especially in a pandemic crisis, by placing DTs at the center. Along with other critical government’s actions and individual behaviors during epidemic and pandemic, DTs should be considered as a prerequisite element. DT can connect the government’s policies with individual behaviors, allowing individuals to understand the government’s intentions and a government to help individuals. As seen on the above, DTs can effectively integrate both containment and mitigation strategies. DTs are no longer in the peripheral position of disaster management. They have become the enablers, facilitators, and integrators of containment and mitigation measures and strategies. This conceptual framework is depicted in Figure 6.

We hope the experience of Korea provides a valuable solution to help other countries and communities in combatting COVID-19. The identical application of Korea DTs to another context, especially countries that have different levels of DT capacities and capabilities, may not be possible. Moreover, there might be many other factors that contribute to the successful utilization of DTs in the context of Korea. However, what this Korean case emphasizes is the role of DTs and the responsibility of a government to effectively use DTs in pandemic management. Therefore, in general, to implement DTs in pandemic situations, the following three factors are in need to be considered. Governments need to have strong initiations in R&D, maximize DTs for public communication channels and network, and facilitate public-private partnerships as well as strong stakeholder networks to advance DTs and their field applications.

## List of Abbreviations

AI: Artificial-Intelligence

CBS: Cellular Broadcasting Service

COVID-19: Coronavirus Disease

DTs: Digital Technologies

EISS: Epidemiological Investigation Support System

KCDC: Korea Centers for Disease Control and Prevention

MOIS: Ministry of the Interior and Safety

SQIS: Smart Quarantine Information System

## References

[1] Korea Central Disease Control Headquarters. Coronavirus Disease-19, Republic of Korea. Ministry of Health and Welfare Cases in Korea Web site. http://ncov.mohw.go.kr/en/. Accessed June 12, 2020.

[2] Majeed A, Seo Y. Can the UK emulate the South Korean approach to COVID-19? BMJ. 2020; 369: 2084 DOI: 10.1136/bmj.m2084 32467112

[3] World Health Organisation. Pandemic influenza preparedness and response: A WHO guidance document In: Programme WGI, ed. Switzerland: World Health Organisation; 2009. 23741778

[4] Nicoll A, Coulombier D. Europe’s initial experience with pandemic (H1N1) 2009 – mitigation and delaying policies and practices. Euro Surveill. 2009; 14(29): 1–6. DOI: 10.2807/ese.14.29.19279-en 19643049

[5] Hollingsworth D, Klinkenberg D. Mitigation strategies for pandemic influenza A: Balancing conflicting policy objectives. PLoS Comput Biol. 2011; 7(2): 1001076–1001076. DOI: 10.1371/journal.pcbi.1001076 PMC3037387 21347316

[6] Mounier Jack S, Jas R. Progress and shortcomings in European national strategic plans for pandemic influenza. Bulletin of the World Health Organization. 2007; 85(12): 923–929. DOI: 10.2471/BLT.06.039834 18278251 PMC2636305

[7] Anderson R, Heesterbeek H. How will country-based mitigation measures influence the course of the COVID-19 epidemic? The Lancet. 2020; 395(10228): 931–934. DOI: 10.1016/S0140-6736(20)30567-5 PMC7158572 32164834

[8] World Health Organisation. WHO global influenza preparedness plan In: Disease DoC, ed. Switzerland: World Health Organisation; 2005.

[9] Ferguson NM, Cummings D. Strategies for mitigating an influenza pandemic. Nature. 2006; 442(7101): 448–452. DOI: 10.1038/nature04795 16642006 PMC7095311

[10] Ferguson N, Cummings D, Cauchemez S, et al. Strategies for containing an emerging influenza pandemic in Southeast Asia. Nature. 2005; 437(7056): 209–214. DOI: 10.1038/nature04017 16079797

[11] Markel H, Stern A. Nonpharmaceutical Influenza Mitigation Strategies, US Communities, 1918–1920 Pandemic. Emerging Infectious Diseases. 2006; 12(12): 1961–1964. DOI: 10.3201/eid1212.060506 17326953 PMC3291356

[12] Anderson R, Fraser C, Ghani A, et al. Epidemiology, transmission dynamics and control of SARS: the 2002–2013. Philosophical Transactions of the Royal Society of London Series B: Biological Sciences. 2004; 359(1447): 1091–1105. DOI: 10.1098/rstb.2004.1490 15306395 PMC1693389

[13] COVID-19 Coronavirus Pandemic. Dadax Limited 2020 https://www.worldometers.info/coronavirus/worldwide-graphs/ Accessed April 23, 2020. DOI: 10.4324/9781003095590-3

[14] International Monetary Fund. World Economic Outlook. Washington, DC: International Monetary Fund; 2020.

[15] Cabinet Office and Strategic Policy Making Team. Professional policy making for the twenty first century. London: Cabinet Office; 2009.

[16] Heinrich C. Evidence-Based Policy and Performance Management: Challenges and Prospects in Two Parallel Movements. The American Review of Public Administration. 2007; 37(3): 255–277. DOI: 10.1177/0275074007301957

[17] Jennings EJ, Hall J. Evidence-Based Practice and the Use of Information in State Agency Decision Making. Journal of Public Administration Research and Theory. 2011; 22(2): 245–266. DOI: 10.1093/jopart/mur040

[18] Herriott RE, Firestone WA. Multisite Qualitative Policy Research: Optimizing Description and Generalizability. Educational Researcher. 1983; 12(2): 14–19. DOI: 10.3102/0013189X012002014

[19] Yin RK. Case study research and applications: design and methods 6th ed. Singapore: Sage publications; 2017.

[20] The Government of the Republic of Korea. Tackling COVID-19 Health, Quarantine and Economic Measures: Korean Experience. Sejong: Ministry of Economy and Finance; 3 31, 2020.

[21] The Government of the Republic of Korea. Flattening the curve on COVID-19: How Korea responded to a pandemic using ICT. Sejong: Ministry of Economy and Finance; 2020.

[22] Korea Centers for Disease Control and Prevention. Coronavirus Disease 2019: Response Guidelines (for Local Governments). Sejong: Korea Ministry of Health and Welfare; 2020.

[23] Park S, Kim Y-M, Yi S, et al. Coronavirus Disease Outbreak in Call Center, South Korea. Emerging infectious diseases. 2020; 26(8). 10.3201/eid2608.201274 PMC7392450 32324530

